# ⁠Advancing ethical AI in healthcare through interpretability

**DOI:** 10.1016/j.patter.2025.101290

**Published:** 2025-06-13

**Authors:** Yilin Ning, Mingxuan Liu, Nan Liu

**Affiliations:** 1 Centre for Quantitative Medicine, Duke-NUS Medical School, Singapore, Singapore; 2 Duke-NUS AI + Medical Sciences Initiative, Duke-NUS Medical School, Singapore, Singapore; 3 Programme in Health Services and Systems Research, Duke-NUS Medical School, Singapore, Singapore; 4 NUS Artificial Intelligence Institute, National University of Singapore, Singapore, Singapore; 5 Department of Biostatistics and Bioinformatics, Duke University, Durham, NC, USA

## Abstract

Interpretability is essential for building trust in health artificial intelligence (AI), but ensuring trustworthiness requires addressing broader ethical concerns, such as fairness, privacy, and reliability. This opinion article discusses the multilayered role of interpretability and transparency in addressing these concerns by highlighting their fundamental contribution to the responsible adoption and regulation of health AI.

## Main text

Wide adoption of artificial intelligence (AI) in healthcare requires addressing major ethical considerations to ensure responsible and trustworthy implementations. To effectively incorporate AI into clinical workflows, key stakeholders (e.g., clinicians, patients, and healthcare leaders) need to be able to understand and assess how models generate outputs and affect decision-making. Model interpretability is essential for this purpose and is therefore an important factor in building trust in AI-driven healthcare applications. To address these needs, researchers have developed various approaches to enhance AI interpretability—either by designing inherently transparent models or by generating *post hoc* explanations for black-box models—while continuing efforts to improve the transparency and trustworthiness of AI systems.

**Figure 1:**
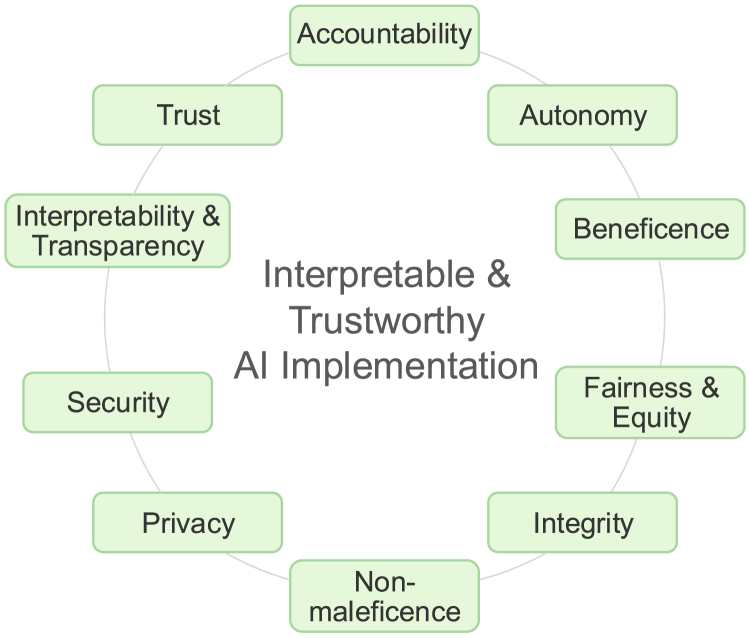
Common ethical principles for responsible health AI applications Accountability refers to clarifying responsibility and establishing safeguards to prevent patient harm from AI use. Autonomy refers to preserving patients’ dignity and rights by ensuring that they have understandable information to make informed choices. Beneficence refers to the putative benefits that AI tools offer and the limits of these benefits. Equity refers to the promotion of fairness in healthcare through the use of AI and equitable access to AI technologies across diverse patient groups. Integrity refers to the commitment to intellectual honesty, responsible conduct, and appropriate acknowledgment in the use of AI. Non-maleficence refers to the prevention of potential risks to patients associated with AI use, such as incorrect or misleading outputs. Privacy refers to the protection of patients’ information from illegitimate access and the confidentiality of personal sensitive information. Security refers to the protection of health data integrity and safety through careful assessment of vulnerabilities in data systems and the prevention of data breaches, cyberattacks, or other threats. Transparency refers to the need for clear disclosure of AI development and evaluation processes and the ability to understand model outputs. Trust refers to the confidence of users in AI to perform prespecified tasks in healthcare, the willingness to accept and integrate AI tools into care delivery, and the recognition of the limitations of AI performance. Detailed definitions of the ethical principles are provided in the scoping review.^1^

The importance of interpretability has made it a recurring theme in ethical discussions and guidelines aimed at ensuring trustworthy AI in healthcare. Meanwhile, these discussions also highlight a broader set of ethical principles that are equally important. For instance, responsible AI should ensure safety, fairness, and privacy by demonstrating adequate performance, avoiding systematic bias, and protecting patient data. Figure 1 visualizes a set of commonly discussed ethical principles summarized in a recent scoping review.^1^ We argue that interpretability and transparency are more than standalone ethical AI principles—they are deeply interconnected with other principles and contribute at multiple levels to build trust in health AI.

In this opinion article, we describe a hierarchy of interpretability and transparency in responsible health AI. This hierarchy begins with developing inherently transparent models and explaining black-box models and expands the focus beyond interpreting a single optimal model to analyzing multiple near-optimal models for more robust insights. It then extends beyond model interpretation to preserving interpretability when addressing broader ethical concerns, such as fairness and privacy. Further, it emphasizes the need for transparent disclosure of model development and evaluation details across the AI life cycle and, ultimately, the open disclosure of risks and mitigation strategies related to a broader set of core ethical principles.

### Enhancing transparency in health AI with interpretability

A central goal of interpretable AI research is to understand how risk predictions are derived from relevant factors and how these factors are selected from a much larger pool of information, both crucial for responsible and trustworthy risk prediction in healthcare. Transparency and interpretability can be achieved at several levels. Simple models, such as logistic regression, decision trees, and scoring models, explicitly show how predictors contribute to risk, making them well suited for healthcare settings, where transparency aids communication between care providers and patients. Recent advancements in interpretable AI have enhanced these traditional approaches. For example, RiskSLIM employs advanced optimization strategies to derive integer-valued coefficients for small subsets of predictors,^2^ and AutoScore develops scoring models based on logistic regression and leverages interpretable AI for efficient variable selection.^3^ GitHub repositories for RiskSLIM and AutoScore offer software implementations along with clinical examples illustrating their use.

Although simplicity and transparency are preferred in healthcare, complex black-box models might be necessary for capturing intricate predictor interactions or analyzing unstructured data, such as images and text. In such cases, *post hoc* explanation methods help to provide some insights into how predictors relate to outcomes. Common tools include local interpretable model-agnostic explanations (LIME) and Shapley additive explanations (SHAP), which approximate predictions by breaking them into additive contributions of each predictor and are not restricted to specific model or data types. SHAP also provides global measures of variable importance by aggregating local explanations. In image analyses, gradient-weighted class activation mapping (Grad-CAM) is widely used to highlight which regions are more relevant to model predictions. These methods are among the most common interpretable AI techniques in clinical use and are integrated into several commercial tools for clinical AI applications.^4^

The methods above, whether building transparent models or explaining black-box ones, often focus on a single model, typically the one with optimal predictive performance. However, relying on a single model can sometimes yield questionable interpretations, for instance, assigning high importance to variables inconsistent with expert knowledge or clinical understanding.^5^ Recent developments extend interpretable AI beyond a single model by exploring the Rashomon set, i.e., the collection of models with near-optimal performance. These models can differ in parameter values, such as variations in regression coefficients in logistic regression, leading to different interpretations of variable importance. Therefore, analyzing a “cloud” of nearly optimal models rather than a single one offers a more comprehensive and robust understanding of variable importance. The variable importance cloud (VIC) framework assesses how variable importance fluctuates across models within the Rashomon set to reveal the range of each predictor’s importance and how changes in reliance on one predictor can be compensated by others.^5^ The Shapley variable importance cloud (ShapleyVIC) builds on this concept by leveraging Shapley values, a widely used method in interpretable AI, to quantify overall importance with an associated uncertainty range, enabling a more statistically robust assessment.^6^ Exploring the Rashomon set not only supports more robust variable selection for transparent models, as exemplified by a study applying ShapleyVIC to clinical risk prediction,^7^ but could also enhance the interpretability of black-box models by offering insights into underlying predictor-outcome relationships.

### Beyond transparency: The role of interpretability in ethical AI

Beyond transparency and interpretability, trustworthy AI needs to align with a broader set of ethical principles, such as the common ones summarized in the recent scoping review^1^ and shown in Figure 1 and the key ethical principles and requirements outlined in the European Union’s framework for trustworthy AI. However, these ethical principles should not be addressed in isolation. Instead, they are deeply interconnected, and we highlight how transparency serves as a foundation for addressing many of them effectively.

Bias is a pressing ethical concern in health AI. AI models should avoid reinforcing historical biases or introducing new ones in future care. Defining bias in health AI is not straightforward, and a common approach measures disparities in model performance across subpopulations (e.g., by sex, race, or ethnicity). Mitigating bias remains a major challenge, partly because many fairness-enhancing techniques rely on complex methods that are difficult to interpret, and their impact on overall predictive performance is not always clear. Several studies have reported that efforts to improve fairness could reduce model performance under certain conditions,^8^ contributing to the widespread perception that a trade-off between fairness and performance is unavoidable. This perception hinders the adoption of fairness-enhancing methods in clinical AI applications. The recent fairness-aware interpretable modeling (FAIM) approach^8^ demonstrated that such a trade-off can be avoided through exploration of the Rashomon set, which enables the analysis of how varying levels of reliance on specific predictors affect fairness within nearly optimal models. This supports the selection of models that improve fairness and interpretability without unnecessary performance loss. In emergency care,^8^ for example, FAIM identified a fairer logistic regression model and revealed predictors potentially linked to clinical bias beyond commonly discussed variables, such as sex and race. Such transparency empowers domain experts in the bias-mitigation process, supports accountability, and increases the likelihood of adoption in practice.

Beyond fairness, data privacy is another critical concern in health AI. Medical research and application inherently require access to sensitive patient data, yet stringent privacy regulations and institutional policies place significant constraints on data sharing. Ensuring data security is particularly challenging when research collaborations span multiple institutions or countries, where diverse populations improve model generalizability but also introduce legal and logistical barriers. Federated learning offers a promising solution by enabling collaborative model development without transferring individual-level data. One common approach in federated learning focuses on optimizing model performance across distributed sites while placing less emphasis on ensuring that the final models remain interpretable. Another approach involves leveraging the statistical properties of transparent models (e.g., regression models) to develop federated prediction models, preserving key statistical properties necessary for conventional inference in healthcare research. Recent advancements have extended the latter approach to develop scoring models through federated frameworks and demonstrated their applicability in emergency care settings,^9^ offering new opportunities for building interpretable AI systems without compromising data privacy. We encourage further research aimed at improving interpretability and transparency within federated learning frameworks for healthcare applications. Doing so will not only enhance privacy protections but also ensure that the resulting models remain clinically meaningful, ethically sound, and suitable for real-world implementation in healthcare.

### From principles to practice: Operationalizing ethical AI with interpretability and transparency

Multiple guidelines, ethical frameworks, and reporting standards have recognized the importance of interpretability and transparency by establishing these principles as core requirements for responsible health AI, including not only prediction models but also emerging technologies, such as generative AI and large language models (LLMs). For instance, interpretability and transparency are central to the European Union’s framework for trustworthy AI, as well as the World Health Organization’s guidance on ethical AI and on large multimodal models. Practical recommendations from the Coalition for Health AI (CHAI) and the ongoing collaborative assessment for responsible and ethical AI implementation (CARE-AI)^10^ further address how and when to operationalize these principles throughout the AI life cycle.

Reporting standards such as the transparent reporting of a multivariable model for individual prognosis or diagnosis (TRIPOD)+AI,^11^ TRIPOD-LLM,^12^ and the transparent reporting of ethics for generative AI (TREGAI) checklist^1^ explicitly emphasize interpretability and transparency among their key ethical principles. Notably, these guidelines and frameworks articulate these concepts across multiple layers. The most direct form, especially for prediction models, involves using interpretable AI methods to explain how predictors contribute to model outputs. Interpretability in this context helps clinicians and other stakeholders understand how predictions are generated, supporting critical evaluation and contributing to trust. Moreover, preserving interpretability plays a key role in addressing other ethical dimensions, as discussed earlier.

Although transparency is sometimes used interchangeably with interpretability, it is a broader construct that extends beyond the relationship between predictors and outcomes. For instance, TRIPOD+AI also promotes transparency by requiring clear reporting of sample selection, predictor choice, modeling methods, and performance assessments.^11^ In the context of LLMs (and multimodal models more broadly), compared with those of TRIPOD+AI, TRIPOD-LLM’s transparency requirements place greater emphasis on aspects such as intended application settings, data sources, model development procedures, evaluation methods, and known limitations.^12^ This layer of transparency enables researchers and healthcare practitioners to critically assess model reliability, identify conditions where the model might perform well or fail, and develop well-founded trust in its use for clinical decision-making, particularly for complex models that lack direct interpretability.

Despite advances in health AI research and applications, meeting transparency and other ethical requirements remains challenging in practice. The TREGAI checklist offers a structured approach to operationalizing transparency and ethical safeguards in research on generative AI in healthcare by providing a template for disclosing risks and mitigation strategies across core ethical dimensions.^1^ This layer of transparent disclosure helps build user trust in the technology and enables the research community to more effectively address its limitations. We strongly advocate for this multilayered integration of interpretability and transparency into the development and deployment of ethical AI to help align AI with ethical principles and effectively communicate such alignment to stakeholders to enhance real-world trust and adoption in healthcare.

### Conclusion

Interpretable AI is essential for ensuring responsible and trustworthy decision-making support in health AI applications. Although significant research has advanced the interpretability of AI prediction models, we advocate for a multilayered integration of interpretability and transparency to address broader ethical challenges that hinder real-world deployment. Beyond explaining AI-driven decision-making processes, we highlight the importance of preserving interpretability when addressing broader ethical considerations, such as bias reduction and privacy preservation. Transparent disclosure of model development, evaluation, and life-cycle management is equally important for promoting responsible use and building trust in health AI. Moreover, in view of the practical difficulties in fully resolving all ethical challenges, openly disclosing risks and mitigating strategies across core ethical principles is essential. Sustained collaboration among AI researchers, healthcare professionals, bioethicists, regulatory bodies, and other key stakeholders is crucial for bridging technical advancements with ethical imperatives and advancing from interpretable AI to responsible and trustworthy AI that drives safe and effective healthcare innovation.

## Acknowledgments

This work was supported by the Duke-NUS Signature Research Programme, funded by the Singapore Ministry of Health. Any opinions, findings, and conclusions or recommendations expressed in this material are those of the authors and do not reflect the views of the Ministry of Health.

## Author contributions

Initial development of ideas, Y.N. and N.L.; drafting of the manuscript, Y.N. and M.L.; critical revision of the manuscript, Y.N., M.L., and N.L.; project oversight: N.L. All authors approved the manuscript and had final responsibility for the decision to submit it for publication.

## Declaration of interests

The authors declare no competing interests.

## Declaration of generative AI and AI-assisted technologies in the writing process

During the preparation of this work, the authors used ChatGPT-4o to improve readability. After using this tool, the authors reviewed and edited the content as needed and take full responsibility for the content of the publication.
